# Homogeneity and Heterogeneity in Social Networks and Brain Aging in Community-dwelling Older Adults in Japan: The NEIGE Study

**DOI:** 10.2188/jea.JE20250534

**Published:** 2026-11-05

**Authors:** Hiroshi Murayama, Shiho Amagasa, Masaki Machida, Shigeru Inoue, Takeo Fujiwara, Yugo Shobugawa

**Affiliations:** 1 Research Team for Social Participation and Healthy Aging, Tokyo Metropolitan Institute for Geriatrics and Gerontology, Tokyo, Japan; 2 Teikyo University School of Public Health, Tokyo, Japan; 3 Department of Preventive Medicine and Public Health, Tokyo Medical University, Tokyo, Japan; 4 Department of Public Health, Institute of Science Tokyo, Tokyo, Japan; 5 Department of Active Aging, Niigata University Graduate School of Medical and Dental Sciences, Niigata, Japan

**Keywords:** social network, homogeneity, heterogeneity, brain volume, older adults

## Abstract

**Background:**

As the global population ages, understanding factors influencing structural brain aging is crucial for dementia prevention. Social networks have emerged as important social factors; however, longitudinal evidence linking specific structural characteristics of social networks to brain volume change remains limited. This study examined whether social network homogeneity and heterogeneity were related to longitudinal changes in brain volume (hippocampus, gray matter, and prefrontal cortex) among community-dwelling older Japanese adults.

**Methods:**

Data were obtained from the Neuron to Environmental Impact Across Generations (NEIGE) study. Participants aged 65–84 years were randomly selected from Tokamachi City, Japan. Of the 527 older adults surveyed at baseline, 279 who underwent magnetic resonance imaging at both baseline (2017) and follow-up (2021) were included. Social relationship (dis)similarities were assessed using ten items, and network homogeneity and heterogeneity domains were derived through factor analysis. Covariates included sociodemographic factors, health behaviors, health conditions, and apolipoprotein E genotype.

**Results:**

Participants were 47.6% male, with a mean age of 73.2 (standard deviation, 5.1) years. Multiple linear regression analyses with inverse probability weighting indicated no association between homogeneous social networks and brain volume changes. However, lower network heterogeneity was significantly associated with a greater decline in hippocampal volume. This was particularly pronounced in the right hippocampus.

**Conclusion:**

Greater social network heterogeneity may attenuate age-related hippocampal volume loss in older adults, highlighting potential benefits of social network heterogeneity for structural brain aging and cognitive aging in later life. Further studies are warranted to clarify the underlying mechanisms.

## INTRODUCTION

With rapid population aging worldwide, increasing attention has focused on factors that influence age-related changes in brain structure among older adults.^1^^–^^4^ Although cognitive decline and dementia represent key clinical outcomes of aging, these conditions are preceded by progressive alterations in brain structure, commonly referred to as structural brain aging. The hippocampus, gray matter, and prefrontal cortex play central roles in memory, cognition, and executive function and are particularly vulnerable to aging and neurodegenerative diseases.^1^^,^^2^^,^^5^^–^^8^ Age-related changes in these regions, particularly hippocampal atrophy, have been closely linked to cognitive decline and disorders such as dementia. Emerging evidence indicates that structural brain aging is shaped not only by neuropathology but also by social and environmental factors across later life. Identifying modifiable social determinants of brain aging is, therefore, critical for mitigating age-related cognitive impairment and preserving brain integrity in older adulthood.^1^^,^^7^ Among these factors, social relationships and social networks have emerged as important determinants that may influence brain aging through interrelated neurobiological, behavioral, and psychosocial pathways, including stress regulation, health-related behaviors, cognitive stimulation, and social engagement.^5^^,^^7^^,^^8^ These pathways, while conceptually distinct, are likely to interact across the life course to shape trajectories of brain aging.

Previous studies suggest that social engagement, including frequency of social contact, is associated with cognitive function and cognitive aging.^1^^,^^2^^,^^4^^–^^8^ However, neuroimaging studies have largely focused on broad indicators, such as social isolation or network size, and have predominantly used cross-sectional designs.^6^^,^^9^^,^^10^ Consequently, it remains unclear whether more nuanced characteristics of social networks, particularly their structural composition, are associated with longitudinal changes in brain volume. A previous Japanese study demonstrated that intra-individual changes in social network diversity were associated with changes in global cognitive function.^11^ However, longitudinal evidence linking social network characteristics to structural brain aging remains limited.

Social networks can be described in terms of homogeneity—when an individual’s relationships are primarily with others who share similar social, economic, and demographic characteristics—and “heterogeneity”—when networks reflect greater diversity in these attributes.^12^^,^^13^ Homogeneous networks are often associated with emotional support and shared social experiences,^14^^,^^15^ whereas heterogeneous networks may provide exposure to diverse perspectives, social roles, and cognitive challenges.^14^^,^^16^ Importantly, both networks have been linked to health and cognitive outcomes in older adults,^17^^–^^20^ suggesting that their potential benefits are not mutually exclusive. However, whether these distinct but overlapping network features are differentially associated with changes in specific brain regions remains unclear. Although Manchella et al examined associations between network characteristics and brain structure in older adults, their cross-sectional design precluded inference about region-specific brain aging trajectories.^9^ Longitudinal evidence is, therefore, needed to determine whether different structural features of social networks are associated with differential patterns of brain volume change over time. As cross-sectional studies cannot distinguish age-related brain changes from pre-existing individual differences in brain structure or social networks, our longitudinal design tracks within-person changes in brain volume and directly links social network characteristics to structural brain aging trajectories.

This longitudinal study of community-dwelling older Japanese adults examined whether social network homogeneity and heterogeneity were associated with changes in brain volume over time. Based on prior theoretical and empirical work suggesting that exposure to diverse social roles and perspectives may involve greater cognitive demands and stimulation,^14^^,^^16^ we hypothesized that higher social network heterogeneity is associated with slower age-related brain atrophy, particularly in regions vulnerable to aging, such as the hippocampus. Concurrently, we did not assume mutually exclusive effects, recognizing that homogeneous networks may also contribute to brain aging through emotional stability and stress buffering. By focusing on specific structural features of social networks and their longitudinal associations with regional brain volume changes, this study extends existing research on social relationships and cognitive aging into the domain of structural brain aging. Our findings contribute to a growing body of literature seeking to identify modifiable social factors that may help preserve brain integrity in later life.

## METHODS

### Study design

This population-based longitudinal cohort study used data from the Neuron to Environmental Impact Across Generations (NEIGE) Study,^21^ which enrolled randomly selected community-dwelling individuals aged 65–84 years in Tokamachi City, Niigata Prefecture, Japan. Tokamachi is an agricultural city located approximately 150 km north of Tokyo and had a population of 54,515 in July 2017, of whom 20,089 (36.9%) were aged ≥65 years. Participants were selected using stratified random sampling by age group (65–74 and 75–84 years) and residential area (downtown and mountainous regions). Individuals with long-term care certification (levels 1–5), hospitalized individuals, or nursing home residents were excluded. In Japan, long-term care certification is part of the national Long-Term Care Insurance system and reflects physical and cognitive functional impairment.^22^ Levels 1–5 indicate functional dependence; individuals at these levels were excluded to restrict the sample to relatively independent community-dwelling older adults and to minimize confounding by disability or cognitive impairment.

A total of 1,346 residents were invited to participate, and 527 completed the baseline survey between September and October 2017 through face-to-face interviews. Baseline brain magnetic resonance imaging (MRI) data were available for 495 participants. All baseline participants were invited to undergo brain MRI as part of the NEIGE study. MRI scans were performed at Niigata Prefectural Tokamachi Hospital by certified radiologic technologists using a standardized protocol. Of the 527 participants, 499 underwent MRI, whereas 28 did not. Reasons were as follows: pacemaker implantation (*n* = 2); intracranial clip placement (*n* = 1); prior MRI at another medical facility (*n* = 1); scheduling conflicts (*n* = 4); declined MRI at the time of scheduling (*n* = 8); and same-day cancellation (*n* = 12). No sedation was required. Four scans were excluded due to substantial image artifacts that could not be adequately corrected, resulting in valid baseline MRI data for 495 participants. In October 2021, 298 participants completed the follow-up survey, of whom 281 underwent follow-up brain MRI, whereas 17 were unable to participate for several reasons: lack of access to transportation to Tokamachi Hospital (*n* = 5); prior MRI at another medical facility (*n* = 2); hospitalization at the time of MRI assessment (*n* = 1); low back pain (*n* = 1); pacemaker implantation (*n* = 1); claustrophobia (*n* = 1); and inability to contact participants (*n* = 6). Valid MRI data from both 2017 and 2021 were available for 279 participants.

The study was approved by the Ethics Committee of Niigata University (approval dates: November 25, 2016 [No. 2666] and November 5, 2021 [No. 2021-0167]). Before the baseline assessment, all participants received detailed information regarding the study objectives, procedures, survey contents, and potential benefits. Written informed consent was obtained from each participant prior to participation.

### Measures

#### Brain volume

Structural brain images were acquired using a 1.5-Tesla MRI system (MAGNETOM Avanto fit; Siemens, Munich, Germany) with a three-dimensional magnetization-prepared rapid gradient-echo protocol. Scanning parameters included a repetition time of 1,700 ms, echo time of 4.31 ms, inversion time of 800 ms, flip angle of 15°, acquisition of 144 contiguous slices with a thickness of 1.25 mm, a field of view of 230 × 230 mm, and a matrix size of 256 × 256. Hippocampal volumes were obtained using automated segmentation with FreeSurfer software (version 6.0; http://surfer.nmr.mgh.harvard.edu). This automated processing pipeline included skull stripping, tissue segmentation, surface reconstruction, and volumetric parcellation, as previously described.^23^ Quality control procedures were conducted by a single trained medical doctor blinded to participants’ characteristics. Visual inspection of FreeSurfer segmentation outputs was performed to identify major errors, including misclassification of brain tissue, inaccurate boundary delineation, and motion-related artifacts. Minor errors were corrected manually according to FreeSurfer guidelines, followed by reprocessing. No independent double-rating was conducted, as all quality control procedures were performed by the same rater following a standardized protocol. MRI scans with substantial artifacts that could not be corrected were excluded from the analysis.

#### Social network homogeneity and heterogeneity

Guided by social network theory, distinguishing ties to socially similar individuals from ties spanning diverse social positions,^12^^,^^13^ homogeneous social networks were defined as networks primarily composed of relationships with individuals sharing similar socio-demographic characteristics (eg, gender, age, or socioeconomic status), whereas heterogeneous social networks were defined as networks including ties differing in these characteristics. To operationalize these constructs, we developed questionnaire items assessing socio-demographic similarities and differences within respondents’ social networks. Item development was informed by semi-structured interviews with 30 community-dwelling older adults who were not participants in the NEIGE study. These interviews explored everyday social interactions to ensure that survey items reflected common social relationship patterns. Based on these interviews, the survey included 11 items capturing interactions with socially similar and dissimilar ties.

Participants were asked whether they had daily interactions with people of the following characteristics relative to themselves: (1) same gender, (2) different gender, (3) older generation, (4) same generation, (5) younger generation, (6) similar socioeconomic position, (7) dissimilar socioeconomic position, (8) similar household composition, (9) dissimilar household composition, (10) similar backgrounds, and (11) dissimilar backgrounds. Responses were recorded on a five-point Likert scale (1 = disagree, 2 = somewhat disagree, 3 = neither, 4 = somewhat agree, or 5 = agree). Interactions with cohabiting family members were included if participants perceived them as part of their daily social relationships. The survey did not explicitly distinguish between face-to-face and online or virtual interactions.

Exploratory factor analysis using maximum likelihood estimation with varimax rotation was conducted on baseline data to identify underlying dimensions of the social network items. Factor retention was based on eigenvalues >1.0, inspection of the scree plot, and interpretability. One item (interactions with older generations) was excluded because of cross-loadings (factor loadings ≥0.30 on multiple factors). The final solution yielded a two-factor structure corresponding to social network homogeneity and heterogeneity (Table 1). Factor scores were calculated by summing the relevant items (range, 5–25). Sum scores were used to enhance interpretability and because internal consistency was acceptable (Cronbach’s α = 0.70 for homogeneous networks and 0.72 for heterogeneous networks). These indicators were divided into tertiles based on the score distribution: low (T1), middle (T2), and high (T3). For network homogeneity, score ranges were 5–18 (low), 19–22 (middle), and 23–25 (high); for network heterogeneity, score ranges were 5–14 (low), 15–17 (middle), and 18–25 (high). Although varimax rotation was used, the two factor scores were moderately correlated (Pearson’s *r* = 0.44), indicating related but distinct dimensions rather than mutually exclusive constructs.

**Table 1.** Factor analysis of social network items

	Factor 1	Factor 2
Network homogeneity		
Interactions with people with similar socioeconomic position	0.82	−0.01
Interactions with people with similar household composition	0.54	0.18
Interactions with people in same generation	0.48	0.23
Interactions with people with the same gender	0.46	0.25
Interactions with people in s similar backgrounds	0.45	0.12
Network heterogeneity		
Interactions with people with dissimilar socioeconomic position	−0.01	0.76
Interactions with people with dissimilar backgrounds	0.14	0.59
Interactions with people in dissimilar household composition	0.22	0.56
Interactions with people with the different gender	0.25	0.50
Interactions with people in younger generation	0.28	0.42
Eigenvalue	3.25	1.55
% of variance explained	32.52	15.49

The values represent factor loadings.

Varimax rotation was performed using the maximum likelihood method.

#### Covariates

Covariates were selected a priori based on previous literature on brain aging and social relationships and their potential role as confounders in the association between social networks and brain volume changes.^4^^–^^8^ Socio-demographic variables included sex (male or female), age, marital status (married or unmarried), employment status (employed or unemployed), educational attainment (≥10 or ≤9 years), and annual household income (<2.5, 2.5–3.9, 4.0–6.9, ≥7.0 million yen, or unknown). Health behaviors included smoking status (currently smoking or not smoking), alcohol consumption (≥5 days or ≤4 days a week), objectively measured physical activity, and body mass index (BMI). Total volume of moderate-to-vigorous physical activity was assessed using a validated accelerometer (Active style Pro HJA-750C; Omron Healthcare, Kyoto, Japan) worn during waking hours for 7 consecutive days.^24^ BMI was calculated as weight divided by height squared (kg/m^2^), and divided into three categories: underweight (<18.5 kg/m^2^), normal weight (18.5–24.9 kg/m^2^), and overweight (≥25.0 kg/m^2^).

Health conditions included self-reported hearing difficulties, physician-diagnosed hypertension, hyperlipidemia, heart disease, stroke, and diabetes mellitus, depressive mood, and cognitive function. Depressive mood was assessed using the 15-item Geriatric Depression Scale (GDS),^25^ which showed good internal consistency (Cronbach’s α = 0.78); a score ≥6 indicated depressive mood. Cognitive function was measured using the Japanese version of the Mini-Mental State Examination (MMSE-J),^26^ which yields scores from 0–30, with lower scores indicating greater cognitive impairment. Genetic factors were assessed using apolipoprotein E (APOE) genotype.^27^ APOE ε4 carrier status was included to account for genetic susceptibility to brain atrophy and cognitive decline, rather than to examine gene–environment interactions, for which the study was not powered.

Although the study focused on modifiable social factors, not all dementia risk factors identified by the Lancet Commission on Dementia Prevention, Intervention, and Care^28^ were available. Nevertheless, several key modifiable factors highlighted by the Commission, such as physical activity, vascular risk factors, depressive mood, and hearing difficulties, were incorporated into the analyses.

### Statistical analysis

Multiple linear regression analyses were conducted to examine the associations between social network homogeneity/heterogeneity and longitudinal changes in brain volume. Outcome variables included changes in hippocampal, gray matter, and prefrontal cortex volumes between baseline (2017) and follow-up (2021). Three models were specified. Model 1 examined the association between social network homogeneity and brain volume changes, adjusting for the covariates and baseline brain volume of the corresponding region. Model 2 examined the association between social network heterogeneity and brain volume changes using the same set of covariates. Model 3 simultaneously included both social network homogeneity and heterogeneity as exposure variables to examine their independent associations with brain volume changes. Given the face-to-face interview design, missing data were minimal, and all analyses were conducted using a complete-case approach. Associations were expressed as unstandardized regression coefficients (b) together with their 95% confidence intervals (CIs). All statistical analyses were conducted using IBM SPSS 29 (IBM Corp., Armonk, NY, USA).

Data from the 2017 and 2021 surveys were analyzed, excluding older adults who did not participate in the 2021 survey. To address potential selection bias, inverse probability weighting was applied using propensity scores estimated via logistic regression, based on the aforementioned covariates and the estimated intracranial volume.

## RESULTS

Table 2 summarizes the baseline demographic, health, and social characteristics of the 279 participants. Males were 47.6%, and the mean age was 73.2 (standard deviation, 5.1) years. Most participants were married (81.1%), and 41.5% were employed. Regarding health behaviors, 8.6% were current smokers, and 36.8% consumed alcohol on ≥5 days per week. In addition to overall sample characteristics, Table 2 presents distributions stratified by tertiles of social network homogeneity and heterogeneity.

**Table 2.** Baseline characteristics of participants

		Total	Network homogeneity			Network heterogeneity		
	
			Low	Middle	High	Low	Middle	High
Sex	Male	47.6	53.9	40.8	52.1	42.2	39.7	60.2
Age, years		73.2 (5.1)	73.3 (4.9)	73.1 (5.1)	73.0 (5.1)	73.4 (5.0)	72.5 (4.8)	73.3 (5.1)
Marital status	Married	81.1	81.6	80.1	82.0	84.9	73.2	81.9
Employment status	Employed	41.5	45.4	37.1	45.0	34.9	46.5	46.2
Education	≤9 years	36.1	31.0	40.8	32.9	38.7	32.3	34.7
Annual household income	<2.5 million yen	27.9	28.2	27.7	28.4	24.6	27.0	32.7
	2.5–3.9 million yen	26.6	33.1	25.2	22.7	30.5	28.6	21.6
	4.0–6.9 million yen	25.6	22.5	25.7	28.4	22.5	27.8	27.5
	≥7.0 million yen	14.0	8.5	18.8	10.6	14.4	13.5	12.3
	Unknown	6.0	7.7	2.5	9.9	8.0	3.2	5.8
Smoking	Currently smoking	8.6	14.1	6.9	5.7	5.9	8.7	11.7
Alcohol consumption	≥5 days a week	36.8	36.9	35.1	40.0	30.8	28.3	50.3
Total volume of moderate-to-vigorous physical activity, METs-hours/day		3.1 (2.3)	3.0 (2.5)	3.1 (2.2)	3.1 (2.4)	2.9 (2.3)	3.1 (2.7)	3.3 (2.1)
Body mass index	Underweight (<18.5 kg/m^2^)	6.8	6.4	7.4	7.1	9.2	2.4	7.1
	Normal weight (18.5–24.9 kg/m^2^)	74.7	72.3	74.3	76.4	74.6	76.2	74.1
	Overweight (≥25.0 kg/m^2^)	18.5	21.3	18.3	16.4	16.2	21.4	18.8
Self-reported hearing difficulties	Yes	24.3	29.1	20.8	22.9	25.8	15.0	28.1
Hypertension	Yes	45.6	44.4	50.7	41.0	50.3	45.7	41.5
Hyperlipidemia	Yes	35.2	34.5	35.1	37.1	31.2	39.7	37.1
Heart disease	Yes	6.9	9.9	5.4	5.8	5.9	11.8	4.1
Stroke	Yes	2.5	2.1	3.0	2.1	3.2	0.8	2.4
Diabetes mellitus	Yes	10.3	14.9	5.0	12.9	10.8	7.9	11.8
Depressive mood	A score of ≥6 in GDS	16.3	31.7	15.3	2.9	20.4	19.7	10.0
Cognitive function	A score of MMSE-J (range: 0–30)	27.1 (2.6)	26.9 (2.9)	27.4 (2.1)	26.9 (2.6)	27.1 (2.7)	27.1 (2.3)	27.1 (2.5)
Apolipoprotein E genotype	ε4	14.8	14.9	15.8	12.1	15.1	7.9	18.2
Estimated intracranial volume, mm^3^		1,432,711.8 (145,753.3)	1,444,296.4 (148,006.8)	1,424,007.5 (149,048.6)	1,428,293.8 (136,965.1)	1,431,541.3 (145,393.2)	1,409,445.3 (160,296.6)	1,446,994.6 (131,623.9)
Total hippocampal volume, mm^3^		7,251.6 (716.9)	7,242.3 (733.9)	7,292.9 (725.3)	7,227.6 (681.6)	7,276.6 (684.0)	7,214.4 (780.8)	7,273.5 (698.5)
Total gray matter volume, mm^3^		570,099.8 (45,141.8)	572,668.0 (44,543.2)	571,640.2 (47,697.8)	564,728.1 (42,229.5)	569,431.0 (43,991.9)	567,237.2 (48,795.3)	572,509.0 (44,073.9)
Total prefrontal cortex volume, mm^3^		118,007.0 (11,214.1)	118,486.4 (10,963.0)	118,341.7 (11,596.2)	116,844.5 (10,993.7)	117,738.5 (10,672.6)	117,819.4 (12,528.0)	118,280.2 (10,884.3)

GDS, Geriatric Depression Scale; MET, metabolic equivalent of task; MMSE-J, Japanese version of the Mini-Mental State Examination; SD, standard deviation.

Values are calculated using inverse probability weighting.

eTable 1 presents descriptive statistics for changes in brain volume and their variation across levels of social network homogeneity and heterogeneity. Changes in total hippocampal, total gray matter, and prefrontal cortex volumes did not differ significantly across low, middle, and high levels of network homogeneity. For network heterogeneity, a trend toward smaller declines in hippocampal volume was observed with higher heterogeneity (*P* = 0.076), whereas no significant differences were found for total gray matter or prefrontal cortex volumes.

Table 3 presents multivariable regression results for associations between social network homogeneity and heterogeneity and changes in total hippocampal volume. Network homogeneity was not associated with hippocampal volume change in models 1 or 3. In contrast, lower social network heterogeneity was associated with greater hippocampal volume decline (*P* for trend = 0.036 in model 2 and 0.048 in model 3). Participants in the lowest heterogeneity tertile showed a significantly greater decline than those in the highest tertile in Model 2 (b = −55.8; 95% CI, −107.4 to −4.3). This association remained after adjustment for network homogeneity in Model 3 (b = −57.3; 95% CI, −113.2 to −1.5).

**Table 3.** Association between the homogeneity and heterogeneity of social networks and changes in total hippocampal volume

	Model 1	Model 2	Model 3
	b (95% CI)	b (95% CI)	b (95% CI)
Homogeneous social network			
Low	−25.4 (−84.4 to 33.7)		5.4 (−60.7 to 71.6)
Middle	−22.8 (−76.2 to 30.7)		−7.0 (−62.4 to 48.4)
High	Ref.		Ref.
*P* for trend	*P* = 0.394		*P* = 0.908
Heterogeneous social network			
Low		−55.8 (−107.4 to −4.3)	−57.3 (−113.2 to −1.5)
Middle		−49.9 (−107.1 to 7.2)	−52.1 (−115.3 to 11.1)
High		Ref.	Ref.
*P* for trend		*P* = 0.036	*P* = 0.048

b, non-standardized regression coefficient; CI, confidence interval.

Values are calculated using inverse probability weighting.

Adjusted for sex, age, marital status, employment status, years of education, annual household income, smoking, alcohol consumption, moderate-to-vigorous physical activity, body mass index, self-reported hearing difficulties, hypertension, hyperlipidemia, heart disease, stroke, diabetes mellitus, depressive mood, cognitive function, apolipoprotein E genotype, and hippocampal volume at baseline.

eTable 2 presents the regression-adjusted mean total hippocampal volumes at baseline and follow-up, along with adjusted changes between time points, stratified by tertiles of social network heterogeneity. For interpretability, percent change relative to baseline volume was calculated. The decline was 4.7% in the low-heterogeneity group and 4.6% in the middle-heterogeneity group, compared with 3.9% in the high-heterogeneity group.

The association between social network characteristics and changes in total gray matter volume is presented in Table 4. No significant trends were observed across tertiles of network homogeneity or heterogeneity. Adjustment for baseline gray matter volume and other covariates did not reveal significant associations. Similarly, no significant associations were observed between either network characteristic and changes in total prefrontal cortex volume (Table 5). Regression coefficients showed no significant trends across categories in any model.

**Table 4.** Association between homogeneity and heterogeneity of social networks and changes in total gray matter volume

	Model 1	Model 2	Model 3
	b (95% CI)	b (95% CI)	b (95% CI)
Homogeneous social network			
Low	2,042.5 (−463.2 to 4,548.3)		2,974.8 (−163.2 to 5,786.3)
Middle	1,292.1 (−980.9 to 3,565.0)		1,763.7 (−597.1 to 4,123.7)
High	Ref.		Ref.
*P* for trend	*P* = 0.109		*P* = 0.065
Heterogeneous social network			
Low		−701.3 (−2,902.5 to 1,499.8)	−1,654.4 (−4,025.6 to 716.8)
Middle		−463.1 (−2,902.0 to 1,975.8)	−1,657.5 (−4,339.5 to 1,024.6)
High		Ref.	Ref.
*P* for trend		*P* = 0.533	*P* = 0.203

b, non-standardized regression coefficient; CI, confidence interval.

Values are calculated using inverse probability weighting.

Adjusted for sex, age, marital status, employment status, years of education, annual household income, smoking, alcohol consumption, moderate-to-vigorous physical activity, body mass index, self-reported hearing difficulties, hypertension, hyperlipidemia, heart disease, stroke, diabetes mellitus, depressive mood, cognitive function, apolipoprotein E genotype, and total gray matter volume at baseline.

**Table 5.** Association between the homogeneity and heterogeneity of social networks and changes in total prefrontal cortex volume

	Model 1	Model 2	Model 3
	b (95% CI)	b (95% CI)	b (95% CI)
Homogeneous social network			
Low	2,057.7 (−598.8 to 4,714.2)		2,465.8 (−507.4 to 5,439.0)
Middle	−951.8 (−3,356.5 to 1,453.0)		−797.4 (−3,287.8 to 1,692.9)
High	Ref.		Ref.
*P* for trend	*P* = 0.138		*P* = 0.292
Heterogeneous social network			
Low		1,595.0 (−738.8 to 3,928.7)	846.4 (−1,664.9 to 3,357.6)
Middle		−944.4 (−3,534.6 to 1,645.8)	−1,945.2 (−4,787.1 to 896.6)
High		Ref.	Ref.
*P* for trend		*P* = 0.161	*P* = 0.349

b, non-standardized regression coefficient; CI, confidence interval.

Values are calculated using inverse probability weighting.

Adjusted for sex, age, marital status, employment status, years of education, annual household income, smoking, alcohol consumption, moderate-to-vigorous physical activity, body mass index, self-reported hearing difficulties, hypertension, hyperlipidemia, heart disease, stroke, diabetes mellitus, depressive mood, cognitive function, apolipoprotein E genotype, and total prefrontal cortex volume at baseline.

As a sensitivity analysis, we used the number of homogeneous and heterogeneous social networks instead of the original network homogeneity and heterogeneity scores. These counts were based on responses of 4 (somewhat agree) or 5 (agree), ranged from 0–5 for each homogeneous/heterogeneous network, and were categorized into tertiles. The results, presented in eTable 3, were consistent with the main findings shown in Table 3, Table 4, and Table 5.

eTable 4 presents associations stratified by right and left hippocampal and prefrontal cortex volumes. In heterogeneous networks, participants with low and moderate levels of heterogeneity exhibited significant reductions in right hippocampal volume compared with those with high heterogeneity (b = −53.6; 95% CI, −85.0 to −22.2, and b = −82.4; 95% CI, −118.0 to −46.8, respectively; *P* for trend = 0.005). No significant trends were observed for left hippocampal or prefrontal cortex volumes in either network type. In homogeneous social networks, no significant associations were observed for hippocampal or prefrontal cortices.

## DISCUSSION

This study examined longitudinal associations between social network diversity (ie, homogeneity and heterogeneity) and brain atrophy in community-dwelling older adults. To our knowledge, this is the first longitudinal investigation of this relationship. The longitudinal design is a major strength, as it enabled assessment of how social network diversity relates to changes in brain volume over time, thereby clarifying temporal associations. Furthermore, the integration of brain MRI with detailed assessments of social network characteristics strengthened the robustness of our results.

Lower social network heterogeneity was associated with greater hippocampal volume decline, with effects more pronounced in the right hippocampus rather than the left. This suggests that network heterogeneity may be particularly relevant to brain regions involved in memory and spatial processing. The hippocampus plays a central role in memory and spatial navigation, and hippocampal atrophy is a hallmark of cognitive aging. The right hippocampus is more strongly associated with spatial memory, whereas the left hippocampus is more closely linked to verbal memory. One potential explanation is that maintaining heterogeneous social networks requires engagement in spatial and contextual processing, such as navigating interactions across different social roles, settings, and environments.^29^ Social interactions have been conceptualized within a “social navigation” framework, whereby individuals map and navigate social contexts using cognitive processes analogous to spatial navigation. These processes may involve hippocampal systems related to spatial memory, with evidence suggesting a particular role of the right hippocampus in navigation and route-based processing.^30^^,^^31^ Moreover, no significant association was found between the homogeneity or heterogeneity of social networks and changes in brain volume in the gray matter or prefrontal cortex. This pattern suggests that the influence of social network diversity on brain health may be more pronounced in regions sensitive to experiential and environmental complexity, such as the hippocampus, a view that is consistent with previous longitudinal neuroimaging studies reporting stronger associations with hippocampal change than with widespread cortical atrophy.^10^

The concept of social capital provides a useful framework for interpreting these findings. Social capital refers to resources embedded within social networks that individuals can access and mobilize.^32^ It is commonly distinguished into two components: bonding social capital, which strengthens shared identities within inward-looking networks, and bridging social capital, which connects individuals across different social groups in outward-looking networks.^33^ From this perspective, homogeneous and heterogeneous social networks may represent complementary, rather than mutually exclusive, dimensions of social relationships. Individuals may benefit simultaneously from emotionally supportive ties with similar others and from cognitively or socially stimulating interactions with dissimilar others. However, this study did not directly measure social capital or underlying neurobiological mechanisms; therefore, the observed associations should be interpreted as theoretically informed rather than causal. Several interacting psychosocial processes may underlie these associations. Prior studies have linked greater social bridging to larger brain volumes, including the hippocampus,^9^ and theories of neuroplasticity suggest that exposure to diverse social interactions may promote brain adaptation and cognitive resilience in later life.^14^^,^^16^ These interpretations remain speculative and warrant further investigation using direct measures of social capital, network dynamics, and biological markers.

Cognitive engagement represents another plausible mechanism linking social network heterogeneity to reduced brain volume decline.^34^ Interacting with socially diverse individuals requires processing varied information, perspectives, and emotional cues, which may provide sustained cognitive stimulation. Such stimulation has been proposed to promote neuroplasticity and preserve brain structures, including the hippocampus. Previous studies have reported associations between socially enriching activities and larger hippocampal volume.^35^^–^^37^ In addition, psychosocial factors such as emotional support, life satisfaction, and mental well-being may also mediate the relationship between social networks and brain health. Stronger social support is associated with lower stress levels, which may protect against cognitive decline.^11^ Furthermore, emotionally rich social interactions may buffer against the adverse effects of social isolation,^38^ a recognized risk factor for cognitive decline and neurodegeneration.

Several limitations should be considered. First, the follow-up period (2017–2021) partly overlapped with the coronavirus disease 2019 (COVID-19) pandemic, and pandemic-related restrictions on social interaction may have influenced both social networks and brain aging during the latter part of follow-up. As a result, the findings may not fully reflect associations under normal conditions. Second, selection bias is possible, as participants who completed both surveys and MRI assessments tended to be healthier and more socioeconomically advantaged than non-participants. Concerns about COVID-19 infection may also have influenced follow-up participation. Although inverse probability weighting was used to address attrition-related bias, residual bias and limited representativeness of the imaging sample cannot be fully excluded. Third, despite the longitudinal design, residual confounding remains possible, and causal inferences should be made cautiously. Unmeasured or imperfectly measured factors related to both social networks and brain aging may have influenced the observed associations. Fourth, measures of social network homogeneity and heterogeneity were derived from exploratory factor analysis of newly developed, self-reported items and may be subject to reporting bias; further validation in independent samples is warranted. Fifth, the analysis focused on structural MRI measures and did not directly assess cognitive outcomes, limiting inference regarding functional implications. Finally, the study was conducted in a rural Japanese community, and social network structures may differ across cultural or societal contexts, which may limit generalizability. Caution is, therefore, required when generalizing the findings to non-Japanese or more urban populations.

### Conclusion

This longitudinal study suggests that lower social network heterogeneity is associated with greater hippocampal atrophy in later life. As social networks tend to become smaller and less diverse with age,^39^ particularly in later life,^40^ maintaining opportunities for socially diverse connections may be relevant for supporting cognitive health among older adults. These findings underscore the potential importance of social network diversity in brain aging, while further studies are warranted to clarify causal mechanisms and to determine whether interventions aimed at enhancing social network heterogeneity can contribute to the prevention of cognitive decline and dementia.

## SUPPLEMENTARY MATERIAL

eTable 1. Changes in brain volume by network homogeneity and heterogeneity

eTable 2. Regression-adjusted mean of total hippocampal volumes by tertiles of social network heterogeneity

eTable 3. Association between the numbers of homogeneity and heterogeneity social networks and changes in total hippocampal, total gray matter, and prefrontal cortex volumes (based on model 3)

eTable 4. Association between the homogeneity and heterogeneity of social networks and changes in left/right hippocampal and prefrontal cortex volumes (based on model 3)
