# Supplementary material for: Homogeneity and Heterogeneity in Social Networks and Brain Aging in Community-dwelling Older Adults in Japan: The NEIGE Study
Source: J Epidemiol. 2026 Nov 5;36(11):346–53. doi: 10.2188/jea.JE20250534 (PMC13662266; doi:10.2188/jea.JE20250534)
Supplement: Supplementary file 1 [file je-36-346-s001.pdf]

eTable 1. Changes in brain volume by network homogeneity and heterogeneity

	Network homogeneity				Network heterogeneity			
	Low	Middle	High	*P*	Low	Middle	High	*P*
Change in total hippocampal volume (mm^3)	-325.9 (210.6)	-323.3 (243.2)	-303.1 (255.7)	0.672	-340.0 (233.7)	-330.6 (207.0)	-285.3 (259.9)	0.076
Change in total gray matter volume (mm^3)	-15,038.1 (11,967.0)	-14,900.4 (9,288.9)	-16,626.7 (10,235.9)	0.278	-15,435.8 (11,371.3)	-15,339.9 (8,820.9)	-15,520.0 (10,471.8)	0.989
Change in total prefrontal cortex volume (mm^3)	-1,654.6 (15,451.7)	-3,555.8 (14,818.9)	-1,489.8 (14,973.8)	0.361	-1,440.2 (14,391.6)	-2,956.7 (17,142.8)	-3,034.1 (14,100.1)	0.542

Values represent mean (standard deviation).

Values are calculated using inverse probability weighting.

eTable 2. Regression-adjusted mean of total hippocampal volumes by tertiles of social network heterogeneity

	2017 adjusted mean[a] (SE), mm^3	2021 adjusted mean[a] (SE), mm^3	Adjusted change[b] (SE), mm^3	% change[c]
Low	7,275.1 (26.5)	6,935.4 (28.4)	-339.8 (5.5)	4.7%
Middle	7,210.2 (34.5)	6,878.9 (37.6)	-331.2 (7.1)	4.6%
High	7,276.4 (31.4)	6,991.3 (31.9)	-285.0 (4.4)	3.9%

SE, standard error.

Values are calculated using inverse probability weighting.

[a] Adjusted for sex, age, marital status, employment status, years of education, annual household income, smoking, alcohol consumption, moderate-to-vigorous physical activity, body mass index, self-reported hearing difficulties, hypertension, hyperlipidemia, heart disease, stroke, diabetes mellitus, depressive mood, cognitive function, and apolipoprotein E at baseline.

[b] Adjusted for sex, age, marital status, employment status, years of education, annual household income, smoking, alcohol consumption, moderate-to-vigorous physical activity, body mass index, self-reported hearing difficulties, hypertension, hyperlipidemia, heart disease, stroke, diabetes mellitus, depressive mood, cognitive function, apolipoprotein E, and total hippocampal volume at baseline.

[c] Percent change was calculated descriptively as (adjusted mean in 2021 − adjusted mean in 2017) / adjusted mean in 2017 × 100.

eTable 3. Association between the numbers of homogeneity and heterogeneity social networks and changes in total hippocampal, total gray matter, and prefrontal cortex volumes (based on model 3)

	Total hippocampal volume<br>b (95% CI)	Total gray matter volume<br>b (95% CI)	Prefrontal cortex volume<br>b (95% CI)
Number of homogeneous social network			
Low (0–2)	8.3 (-58.7 to 75.4)	1957.4 (-881.9 to 4,796.7)	-555.5 (-3,600.2 to 2,489.2)
Middle (3–4)	8.1 (-46.4 to 62.6)	1326.8 (-986.2 to 3,639.9)	-1785.9 (-4,257.5 to 685.7)
High (5)	Ref.	Ref.	Ref.
P for trend	*P*=0.921	*P*=0.274	*P*=0.576
Number of heterogeneous social network			
Low (0)	-66.3 (-131.8 to -0.8)	1145.0 (-1,630.9 to 3,921.0)	2307.6 (-657.8 to 5,272.9)
Middle (1–2)	-66.8 (-119.4 to -14.2)	-2045.2 (-4,274.7 to 184.4)	351.1 (-2,042.7 to 2,744.9)
High (3–5)	Ref.	Ref.	Ref.
P for trend	*P*=0.025	*P*=0.676	*P*=0.194

b, non-standardized regression coefficient; CI, confidence interval.

Values are calculated using inverse probability weighting.

Adjusted for sex, age, marital status, employment status, years of education, annual household income, smoking, alcohol consumption, moderate-to-vigorous physical activity, body mass index, self-reported hearing difficulties, hypertension, hyperlipidemia, heart disease, stroke, diabetes mellitus, depressive mood, cognitive function, apolipoprotein E, and each outcome (total hippocampal, total gray matter, and prefrontal cortex volumes) at baseline.

eTable 4. Association between the homogeneity and heterogeneity of social networks and changes in left/right hippocampal and prefrontal cortex volumes (based on model 3)

	Right hippocampal volume b (95% CI)	Left hippocampal volume b (95% CI)	Right prefrontal cortex volume b (95% CI)	Left prefrontal cortex volume b (95% CI)
Homogeneous social network				
Low	32.4 (-4.8 to 69.7)	-26.6 (-66.4 to 13.2)	1,280.9 (-249.3 to 2,811.0)	1,190.0 (-306.7 to 2,686.7)
Middle	11.8 (-19.4 to 42.9)	-17.0 (-50.3 to 16.4)	-322.8 (-1,604.5 to 959.0)	-477.7 (-1,730.8 to 775.5)
High	Ref.	Ref.	Ref.	Ref.
P for trend	*P*=0.543	*P*=0.445	*P*=0.241	*P*=0.367
Heterogeneous social network				
Low	-53.6 (-85.0 to -22.2)	-2.1 (-35.7 to 31.5)	358.1 (-932.6 to 1,648.8)	475.6 (-790.3 to 1,741.5)
Middle	-82.4 (-118.0 to -46.8)	30.1 (-7.9 to 68.1)	-849.4 (-2,311.4 to 612.6)	-1,100.6 (-2,531.4 to 330.2)
High	Ref.	Ref.	Ref.	Ref.
P for trend	*P*=0.005	*P*=0.681	*P*=0.439	*P*=0.294

b, non-standardized regression coefficient; CI, confidence interval.

Values are calculated using inverse probability weighting.

Adjusted for sex, age, marital status, employment status, years of education, annual household income, smoking, alcohol consumption, moderate-to-vigorous physical activity, body mass index, self-reported hearing difficulties, hypertension, hyperlipidemia, heart disease, stroke, diabetes mellitus, depressive mood, cognitive function, apolipoprotein E, and each outcome (left/right hippocampal and prefrontal cortex volumes) at baseline.
